# Tailoring electron bunch quality in laser-plasma acceleration: a comparative study of Bessel-Gaussian and Gaussian laser profiles under variable plasma density geometries

**DOI:** 10.1038/s41598-026-39821-9

**Published:** 2026-02-12

**Authors:** R. Khooniki, R. Fallah, S. M. Khorashadizadeh, A. R. Niknam

**Affiliations:** 1 Department of Physics, University of Birjand, Birjand, Iran; 2 Laser and Plasma Research Institute, Shahid Beheshti University, Tehran, Iran

**Keywords:** Particle-in-cell simulation, Laser wakefield acceleration, Bessel-Gaussian laser pulse, Plasma, Electron bunch quality, Down-ramp injection., Engineering, Physics

## Abstract

Controlling electron injection and beam quality in laser wakefield acceleration (LWFA) requires coordinated manipulation of both the driving laser structure and the plasma density profile. In this work, a systematic particle-in-cell (PIC) study is performed to investigate how longitudinal plasma density tailoring, characterized by the high-density plateau length $L_{\mathrm{high}}$, interacts with laser pulse shaping to regulate electron injection and final beam properties. Gaussian (G) and zeroth-order Bessel–Gaussian (BG) laser pulses are compared under strictly equal total laser-energy conditions using multi-section plasma density profiles. The simulations show that BG pulses promote extended and repeated injection, resulting in higher trapped charge across all investigated values of $L_{\mathrm{high}}$. This enhancement is accompanied by increased beam loading, which reduces the effective accelerating field and limits the attainable peak energy. In contrast, G pulses favor more localized injection with lower trapped charge but higher peak energy and improved spectral quality, particularly when the high-density plateau is removed ($L_{\mathrm{high}} = 0$). Importantly, $L_{\mathrm{high}}$ is identified as a robust and practical control parameter for tuning injection duration and balancing charge–energy trade-offs for both laser drivers. Despite the distinct injection dynamics, no fundamental differences in transverse emittance behavior are observed between G and BG pulses for identical plasma configurations. Instead, the emittance evolution is governed by the timing and spatial localization of injected electrons, as controlled by the tailored density geometry. These results establish a design-oriented framework in which moderate laser pulse shaping, combined with longitudinal plasma density tailoring, provides complementary and experimentally accessible pathways for optimizing beam parameters in LWFA.

## Introduction

Plasma accelerators have emerged as a transformative field over the past few decades, rapidly garnering global scientific interest. Their appeal stems from their inherently compact footprint, significantly lower construction costs compared to conventional counterparts, and the vast potential across applications spanning high-energy physics, advanced medical treatments, and industrial processes^1–5^. The fundamental principle relies on leveraging the plasma’s inherent capacity to sustain electric fields orders of magnitude stronger than those achievable in radiofrequency cavities, a concept pioneered by Tajima and Dawson^6^. This process, known as Laser Wakefield Acceleration (LWFA), involves an intense laser pulse driving a longitudinal plasma wave–the wakefield–whose enormous accelerating gradient can propel charged particles to multi-GeV energies over distances of mere centimeters, vastly exceeding the capabilities of conventional technology^7,8^. For highly relativistic drivers, this wake structure often simplifies into the “bubble regime”^9^, forming a cavity that facilitates high-energy particle injection.

Despite the clear advantage in acceleration gradient, achieving precise control over the accelerated electron bunch quality remains a central challenge. While the most common initial technique, self-injection^10,11^, relies on the stochastic breaking of the plasma wave, this inherent lack of control leads to poor reproducibility in critical beam parameters. This process occurs when the plasma wave, driven by an intense laser pulse, reaches a sufficient amplitude to trap background electrons. Specifically, when the laser propagates through a plasma with a density high enough relative to its parameters, nonlinear effects cause the pulse to evolve, most notably via self-compression. These dynamics steepen the plasma wave until it eventually breaks, allowing electrons from the plasma to cross the separatrix and become trapped in the wakefield, where they are subsequently accelerated. To achieve greater control over the electron injection process, several alternative schemes have been developed, including ionization-induced injection^12–14^, optical injection via colliding laser pulses^15–17^, and density transition-based injection^18–23^. Density down-ramp injection has emerged as a robust and controllable method for electron injection in LWFA, especially for improving bunch quality. As the laser pulse propagates through a density down-ramp, a region where the background electron density decreases along the direction of propagation, the plasma frequency correspondingly decreases. This reduction leads to an expansion of the bubble. During this expansion, some background electrons returning toward the optical axis become trapped within the bubble, resulting in localized injection. This density down-ramp injection scheme was first proposed by Bulanov et al.^24^, who considered a gradual density transition to reduce the phase velocity of the plasma wave, thereby lowering the threshold for electron trapping. A numerical study by Massimo et al.^25^ examined the effects of laser, accompanied by variations in the density transition shape, on density transition injection in the context of LWFA. Simulations performed by Ekerfelt et al.^26^ showed that by adjusting the steepness and magnitude of the density transition, key bunch parameters can be effectively tuned. Recently, Cobo et al.^27^ experimentally and numerically investigated density transition injection in laser wakefield acceleration, demonstrating that tailored plasma density gradients have a significant influence on electron injection and beam quality. In a recent numerical study, A Jain et al.^28^ explored down-ramp injection in LWFA using both a density bump and a single down-ramp.

As the laser propagates through the plasma, its shape evolves due to several nonlinear effects, including group velocity dispersion, self-phase modulation, and self-steepening^29–31^, resulting in an asymmetrical temporal pulse profile. Therefore, one effective strategy involves modifying the laser pulse shape by tailoring its temporal and spatial characteristics to enable more precise control over the wakefield structure, ultimately improving the quality of the electron beam in laser wakefield acceleration^32–37^. Characteristics of laser pulses, such as frequency, electric field amplitude, pulse length, and pulse shape, are key parameters governing the dynamics of laser wakefield acceleration (LWFA) and directly impact the quality of the resulting electron beam^38–44^. Most studies on electron acceleration and wakefield structure have mainly focused on Gaussian laser pulses. However, in recent years, some research related to laser wakefield acceleration (LWFA) has explored the effect of laser pulses with a Bessel-Gaussian profile^45^. Fallah et al.^46^ performed a comparative analysis of BG and G laser pulses, demonstrating that the wakefield generated by the BG laser pulse is stronger than that produced by the G laser pulse under the same laser-plasma interaction parameters. The simulation results obtained by Abedi-Varaki et al.^47^ show the quality of injected electrons with various nitrogen concentrations in LWFA driven by a BG laser pulse. The Bessel beam is a prominent member of the non-diffracting beam family, widely studied for its unique propagation characteristics and deep relevance to both fundamental and applied physics. Its versatile properties have enabled a broad range of applications, including precision cutting, microscale operations, advanced material processing, and medical technologies. Bessel-Gaussian (BG) beams have been experimentally realized using compact optical setups that employ axicons, spatial phase plates (SPPs), spatial light modulators (SLMs), and etc., allowing for precise control over the beam structure^48–50^.

This report focuses on a targeted Particle-in-Cell (PIC) simulation study using the FBPIC code^51^ to decouple and analyze the synergistic effects of laser pulse shape and plasma density profiling on LWFA electron bunches. Specifically, we investigate the comparative performance of the zeroth-order Bessel-Gaussian (BG) and standard Gaussian (G) laser pulses when coupled with systematically adjusted plasma up-ramp and plateau lengths. By fixing all other parameters, this controlled methodology isolates the influence of the tailored density geometry on beam quality metrics. Our initial analysis confirms that, for identical laser pulse energy, the BG pulse achieves a higher peak intensity, thus facilitating a greater injected charge into the bubble. However, a subsequent investigation into post-injection dynamics reveals that the G pulse yields a higher mean electron bunch energy across the acceleration stage. Ultimately, our findings conclusively demonstrate that the applied density profiling approach critically influences emittance, revealing that a lower emittance is observed consistently in the case of the BG laser pulse, thereby establishing a pathway for high-quality beam generation through precise control over the laser-plasma interface.

The remainder of this paper is organized as follows: Section II details the simulation setup, including the physical parameters of the BG and G laser pulses and the FBPIC code configuration. Section III presents the comparative results for charge injection and energy spectra, while Section IV is dedicated to the analysis of beam emittance and the role of the density ramp profile. Finally, Section V concludes the paper with a summary of the key findings and implications for future high-quality LWFA experiments.

**Fig. 1:**
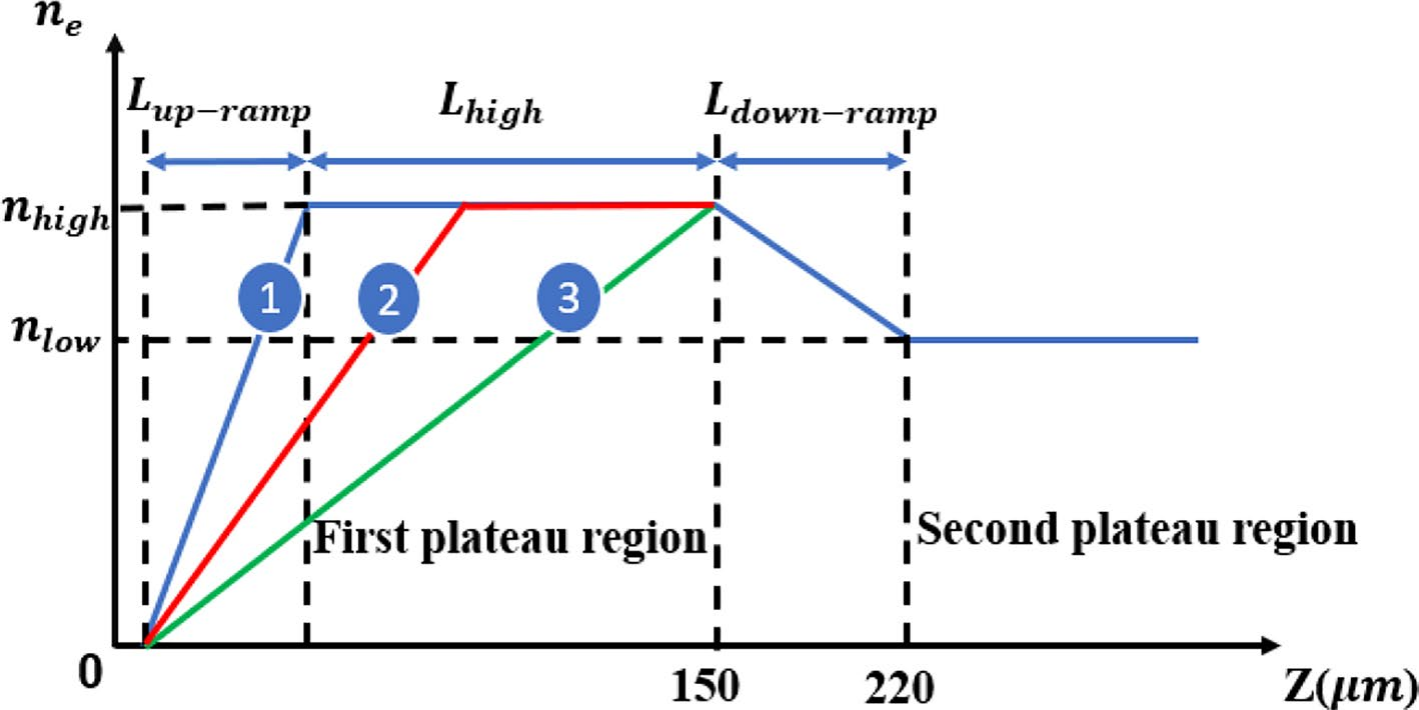
Schematic representation of the plasma density profile employed across the three primary simulation cases. The profile is characterized by an initial up-ramp of fixed length $L_{up-ramp}$, followed by a first plateau region whose length, ${L_{high}}$, is systematically varied: Case 1 (${L_{high}}=100 \upmu m$), Case 2 (${L_{high}}=60 \upmu m$), and Case 3 (where the high-density plateau is eliminated). This initial section is followed by a down-ramp region leading into a final, long plateau, establishing a total plasma channel length of $1500 \upmu m$.

## Simulation model

We utilize Particle-in-Cell (PIC) simulations to explore the operation of LWFA on the electron bunch injected using a density down-ramp. To deepen our understanding of the LWFA process, PIC simulations using the Quasi-3D FBPIC code^51^ are conducted to model laser-plasma interactions. The simulation box has dimensions $(z,r)=(60,60) \upmu m$ with a mesh resolution $\delta z=0.04 \upmu m$ and $\delta r=0.2 \upmu m$ in longitudinal and transverse directions, respectively. A grid convergence test was performed by reducing the transverse grid spacing from $0.2~\upmu$m to $0.15~\upmu$m and $0.12~\upmu$m for a representative case (BG laser pulse with $L_{\mathrm{high}} = 100~\upmu$m). Variations in all key beam parameters remained below 3%, confirming that the chosen resolution is sufficient to accurately capture the relevant physics. A moving window propagating at the speed of light in the longitudinal direction is employed. In our simulations, two azimuthal modes m=0 and 1 are used to perform simulations with (2, 2, 8) particles per cell in each grid size, i.e. $(r,z, \theta )$. Here, we consider linearly polarized Gaussian and zeroth-order Bessel–Gaussian laser pulses with identical energy, propagating along the z-direction through the plasma. The corresponding electric-field distributions are characterized as follows:

1 $$\begin{aligned} & { \vec {E}_G} = {\hat{x}}{E_{0}^{G}}{e^{\left( {\frac{{ - {r^2}}}{{{{w_0} ^2}}} - \frac{{{{(z - ct)}^2}}}{{{c^2}{\tau ^2}}}} \right) }}\cos \left( {k\left( {z - ct} \right) } \right) , \end{aligned}$$

2 $$\begin{aligned} & {\vec {E}_{BG}} = {\hat{x}}{ E_{0}^{BG}}{e^{\left( {\frac{{ - {r^2}}}{{{{w_0} ^2}}} - \frac{{{{(z - ct)}^2}}}{{{c^2}{\tau ^2}}}} \right) }}{J_0}\left( {\frac{{{k_n}}}{w_0 }r} \right) \cos \left( {k\left( {z - ct} \right) } \right) , \end{aligned}$$

Here, $E_{0}^{G}$ and $E_{0}^{BG}$ denote the peak electric field amplitudes of the Gaussian and Bessel–Gaussian laser pulses, respectively. For a fixed total laser energy, these amplitudes are inherently different due to their distinct transverse intensity distributions. The function $J_0$ denotes the zeroth-order Bessel function, and $w_0$ represents the laser beam waist. For the zeroth-order BG pulse, the parameter $k_n = 1.2$ is deliberately chosen such that the central transverse intensity profile remains close to that of a standard G beam, while still preserving the characteristic ring structure of a BG mode. This moderate value ensures a controlled and meaningful comparison between G and BG laser pulses by minimizing differences that arise solely from the transverse profile shape, rather than from the wakefield dynamics under investigation. Moreover, BG beams with similar parameters can be readily generated in experiments using axicon-based optical setups^48–50^. In contrast, $k_n = 0$ corresponds to a standard G laser pulse. The laser parameters, selected based on a Ti:Sa laser system for the simulations, are the wavelength $\lambda = 0.8~\upmu \mathrm{m}$, FWHM pulse duration $\tau _{\mathrm{FWHM}} = 28~\mathrm{fs}$, laser waist $w_0 = 12~\upmu \mathrm{m}$, and normalized vector potential $a_0 = 2.5$ for the G pulse and $a_0 = 2.93$ for the BG pulse, corresponding to equal total pulse energies of $0.9~\mathrm{J}$.

The employed plasma density profile, illustrated in Fig. 1, comprises a linear up-ramp of length ${\mathrm{L}}_{{\mathrm{up - ramp}}}$ that transitions the density from vacuum to a high-density plateau, ${n_{high}}=6{\mathrm{\times }}{10^{18}}c{m^{ - 3}}$. This plateau region extends for a length defined by ${n_{high}}$ and is followed by a linear density down-ramp of length ${\mathrm{L}}_{\mathrm{down - ramp}}=70 \upmu m$, which transitions the density to a lower value, ${n_{low}}=4 {\mathrm{\times }}{10^{18}}c{m^{ - 3}}$. It is crucial to note that while the overall plasma parameters were fixed across the experiments, the length of the high-density plateau, ${L_{high}}$, was the primary varied parameter, constrained such that the total distance from the up-ramp entrance to the end of the first plateau remained invariant. Consequently, we investigate three specific cases for ${L_{high}}$: 0, 60 and $100\upmu m$. The resulting characteristics of the injected electron charge are then examined by varying ${L_{high}}$ for both the G and BG laser pulses.

## Results and discussions

Throughout this work, all comparisons between BG and G laser pulses are performed under the strict constraint of equal total laser energy. Under this condition, the peak intensity and effective $a_0$ are not independent tuning parameters but instead emerge from the intrinsic transverse intensity distribution of each pulse shape. This approach enables a physically fair and controlled isolation of pulse-shape effects on wake evolution and electron injection.

**Fig. 2:**
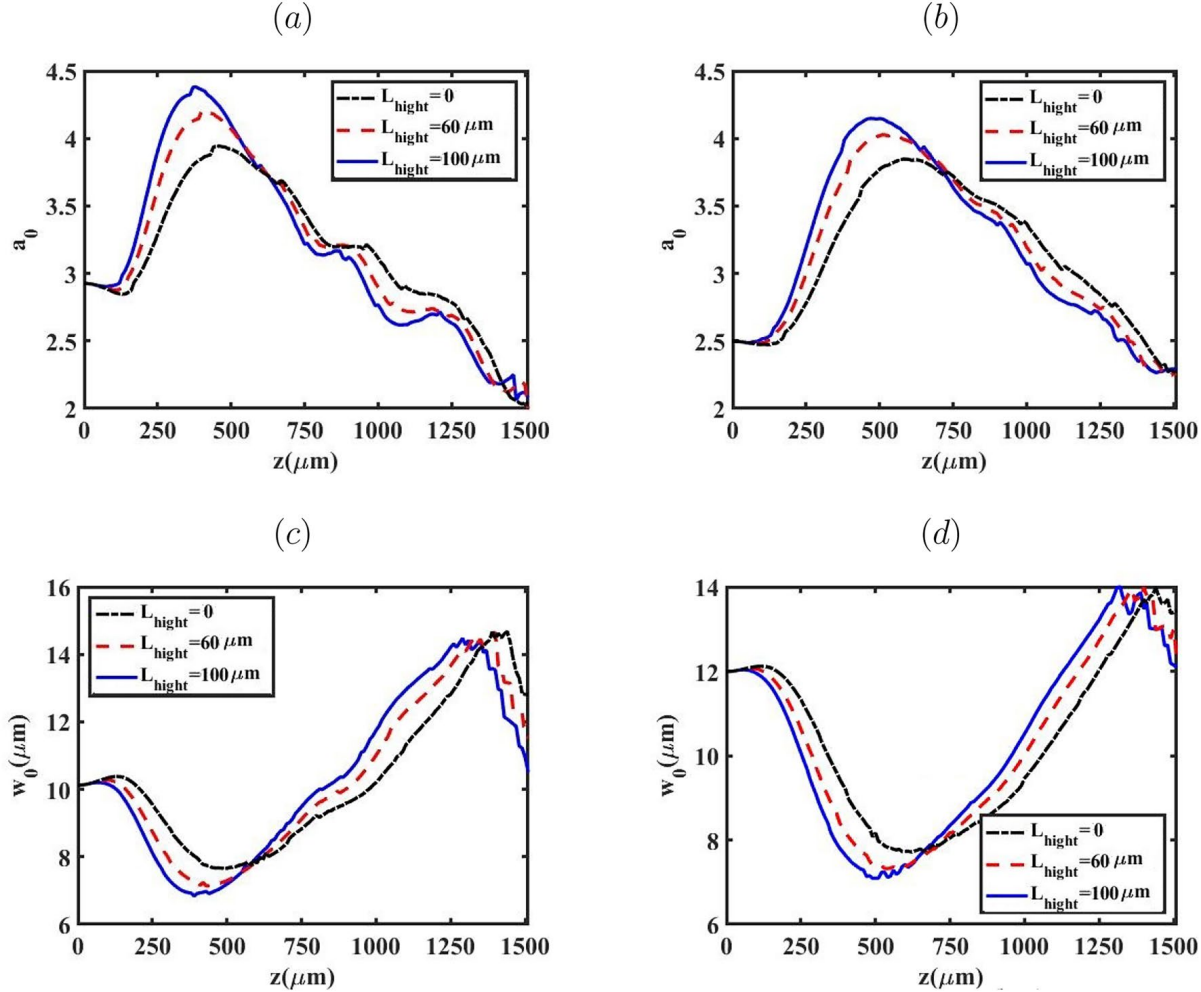
The variation of the laser’s normalized amplitude ($a_0$ ) and waist ($w_0$) as a function of laser peak position for BG (left column) and G (right column) laser pulse.

The evolution of the normalized laser peak potential–which governs the bubble size and hence the electron injection–is examined in Fig. 2 via the simultaneous tracking of the normalized peak intensity $a_0$ and the laser waist $w_0$ along the propagation axis for both BG and G pulses. Variations of the up-ramp length $L_{\text {high}}$ systematically alter the intensity evolution, which in turn modifies the self-focusing dynamics. For $L_{\text {high}}=100~\upmu \text {m}$ the focusing is markedly stronger, a consequence of the steeper up-ramp that rapidly lowers the relativistic self-focusing critical power $P_c = 17.4\,(\omega /\omega _p)^2$ GW, allowing the condition $P>P_c$ to be met earlier. The resulting higher $a_0$ reduces the injection threshold in the subsequent down-ramp, leading to the larger trapped charge observed for the BG profile. Beyond $z \simeq 1250~\upmu \text {m}$, however, the usual inverse relation between $a_0$ and $w_0$ ceases: significant pump-depletion transfers a substantial fraction of the laser energy to the plasma wake, causing $a_0$ to decline even while $w_0$ continues to shrink under residual self-focusing. This pump-depletion regime ultimately limits the further growth of the wake and influences the final energy and charge of the accelerated bunch.

**Fig. 3:**
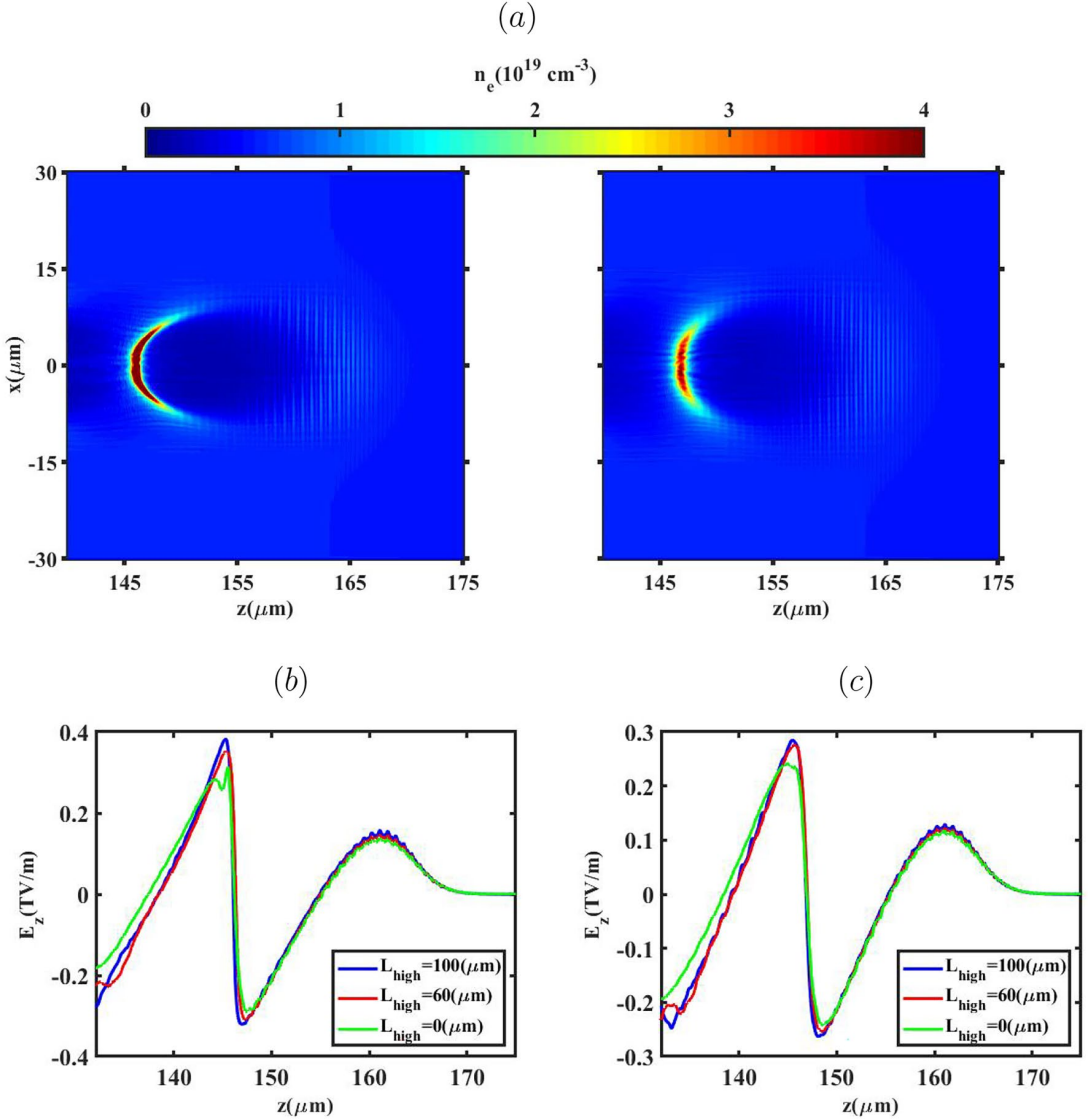
The electron number density distribution (**a**) for the case of ${L_{high}}=100 \upmu m$ (left: BG, right: G), and the corresponding accelerating field ($E_z$) for the BG (**b**) and G (**c**) laser pulses before electron injection.

**Fig. 4:**
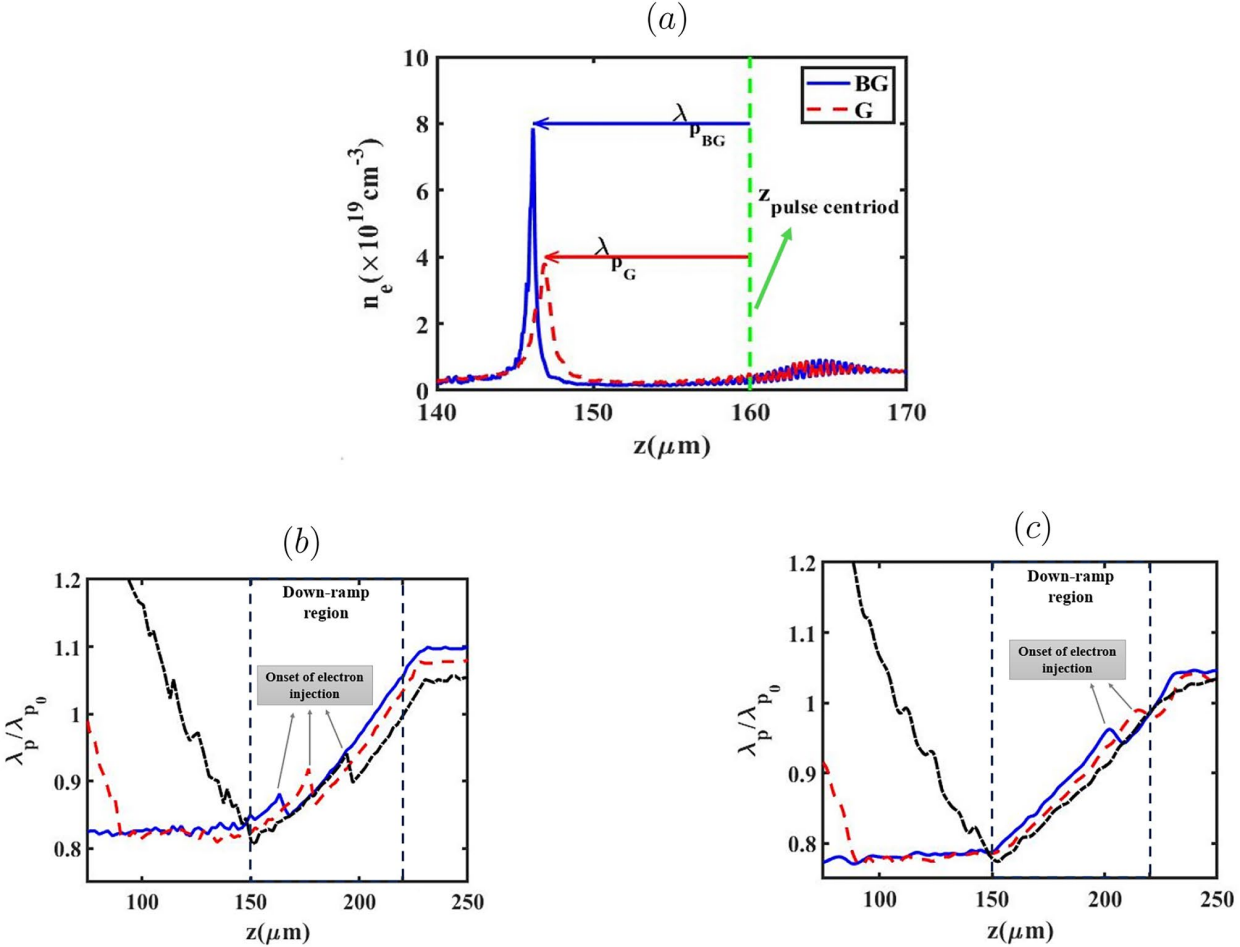
Plots of the electron density distribution for both laser pulses (**a**), and the plasma wavelength variations for all cases of $L_{high}$ (black: ${L_{high}}=0$, red: ${L_{high}}=60 \upmu m$ and blue: ${L_{high}}=100 \upmu m$), when the BG (**b**) and G (**c**) laser pulses propagate through the down-ramp region.

**Fig. 5:**
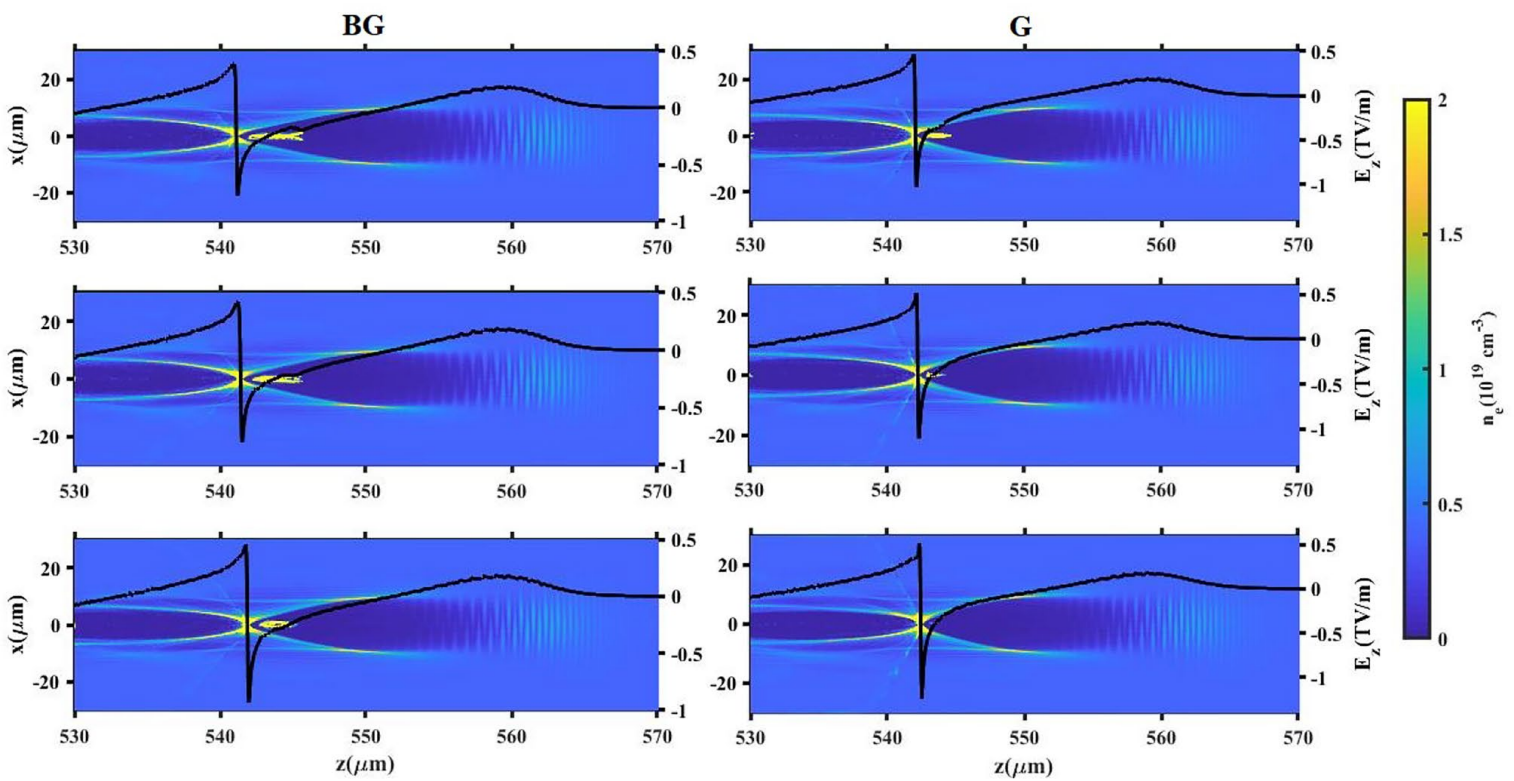
The electron number density distribution and accelerating field ($E_z$) after the down-ramp region for lengths ${L_{high}}$ of $100 \upmu m$, $60 \upmu m$ and 0 (top to bottom), when the BG (left) and G (right) laser pulses propagate through the second plateau region.

Figure 3 displays the plasma density distribution for the case of ${L_{high}}=100 \upmu m$, alongside the wakefields generated by both laser pulses types upon reaching the entrance of the down-ramp region (prior to electron injection). As depicted in Fig. 3a, the BG laser pulse creates a distinct bubble-like structure because the electrons, propelled by the BG laser, experience a stronger transverse ponderomotive force compared to those influenced by the G laser pulse. Consequently, a complete ion cavity is established behind the BG pulse. As indicated in Fig. 3a, electron injection has not yet commenced because the rear portion of the bubble is situated upstream of the down-ramp region. Moreover, Fig. 3 reveals that the size of the bubble structure expands further for the BG laser pulse. This expansion is attributed to the effect of $a_0$^52^, highlighting how the variation in bubble size directly impacts the charge injected as the laser pulse traverses the down-ramp region. It is further observed that the wakefield is more intense for the BG pulse than for the G pulse, owing to the higher momenta gained by the electrons. This is because the number of electrons capable of reaching the bubble rear increases with a higher $a_0$. To understand the influence of the laser pulse shape on the injection process, the evolution of the plasma wavelength (${\lambda _{p}}$) is examined. Figure 4a–c illustrate the lineouts of the electron density distribution (a) for ${L_{high}}=100 \upmu m$, and the evolution of the plasma wavelength for all ${L_{high}}$ cases as the BG (b) and G (c) laser pulses pass through the down-ramp region. The plasma wavelength derived from PIC simulations is defined as the longitudinal distance between the centroid of the laser pulse and the electron density peak at the rear of the bubble. Figure 4a confirms that the BG laser pulse yields a larger bubble structure compared to the G laser pulse. In Fig. 4b,c, ${\lambda _{p_0}}$ denotes the plasma frequency corresponding to the low-density background plasma, $n_{low}$. Within the down-ramp region $(150 \le z(\upmu m) \le 220)$ for both laser pulses, the plasma wavelength, and consequently the bubble, initially expands due to a decrease in plasma frequency. Following this expansion, a subsequent drop occurs where background electrons possessing sufficiently high phase velocity and greater bubble velocity are injected from the rear side of the expanding bubble and become trapped within it. Therefore, during the propagation of the BG laser pulse, the average background electron density in the injection zones is higher than that during the G laser pulse propagation. This difference results in earlier injection and, consequently, a greater quantity of charge being trapped within the bubble for the BG profile. The results indicate that electron bunch injection within the bubble occurs earlier when ${L_{high}}=100 \upmu m$ for both laser types, signifying that a higher charge is injected under this specific condition. The electron density distribution and the on-axis accelerating electric field generated by the BG and G laser pulses, after the electron bunch is injected into the bubble and accelerated in the second plateau region, are depicted in Fig. 5. Our simulation analysis reveals a direct, positive correlation between the final longitudinal bubble size–measured post-injection–and the variation in ${L_{high}}$ across the range of 0 to 100 $\upmu m$. Crucially, the magnitude of the injected charge co-varies directly with this bubble size increase, a dependency that aligns with the theoretical framework established by Ekerfelt et al.^26^. A detailed examination of these plots highlights the significance of the beam loading effect. Specifically, when ${L_{high}}$=0, the G laser pulse propagation is characterized by a negligible beam loading effect, primarily resulting from the insufficient injected charge density of only 0.2 pC. Conversely, the maximum charge loading is achieved in the case of ${L_{high}}=100 \upmu m$ during the propagation of the BG laser pulse. This substantial charge loading manifests physically as a pronounced, non-linear deformation of the longitudinal accelerating electric field profile behind the pulse. For both established laser profiles, the quantified amount of charge successfully trapped and accelerated within the bubble is found to be in robust agreement with the predictive insights derived from our preceding analysis of the injection mechanism.

**Fig. 6:**
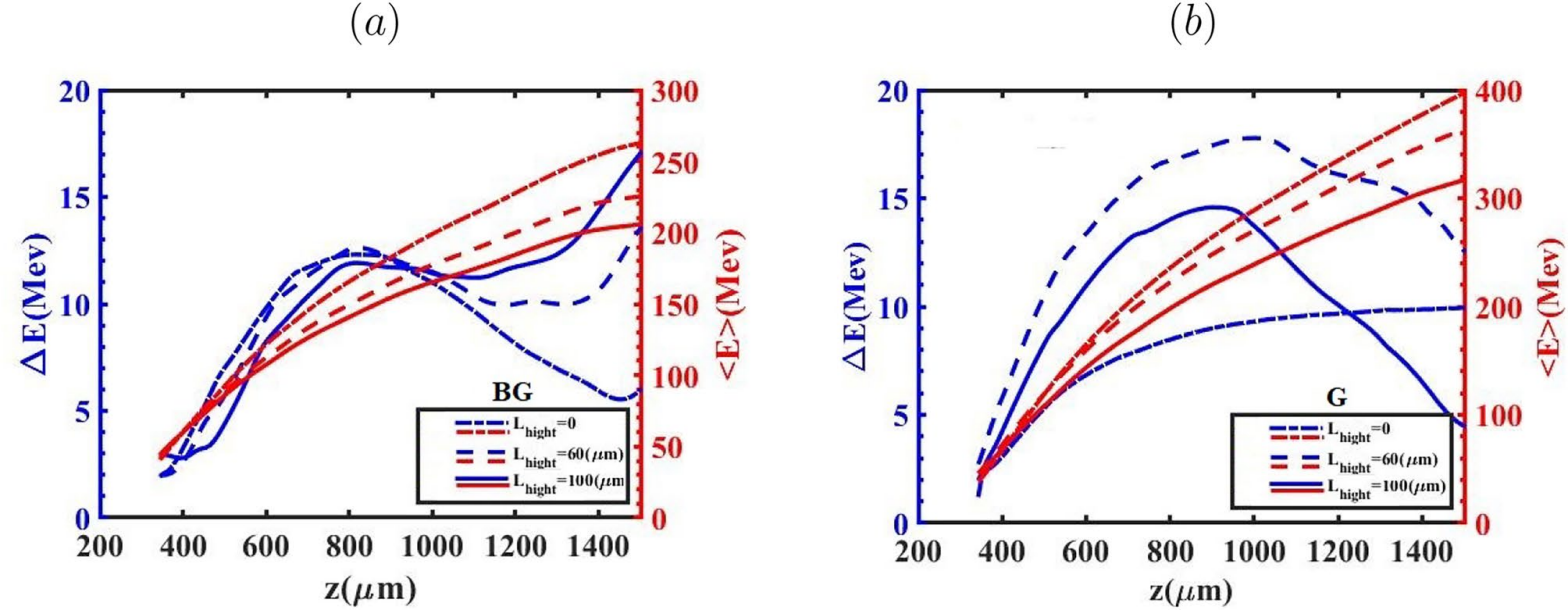
The simulation results show: the evolution of the electron bunch energy spread with respect to propagation distance for all ${L_{high}}$ values, corresponding to the (**a**) BG and (**b**) G laser pulses propagating through the plasma.

**Fig. 7:**
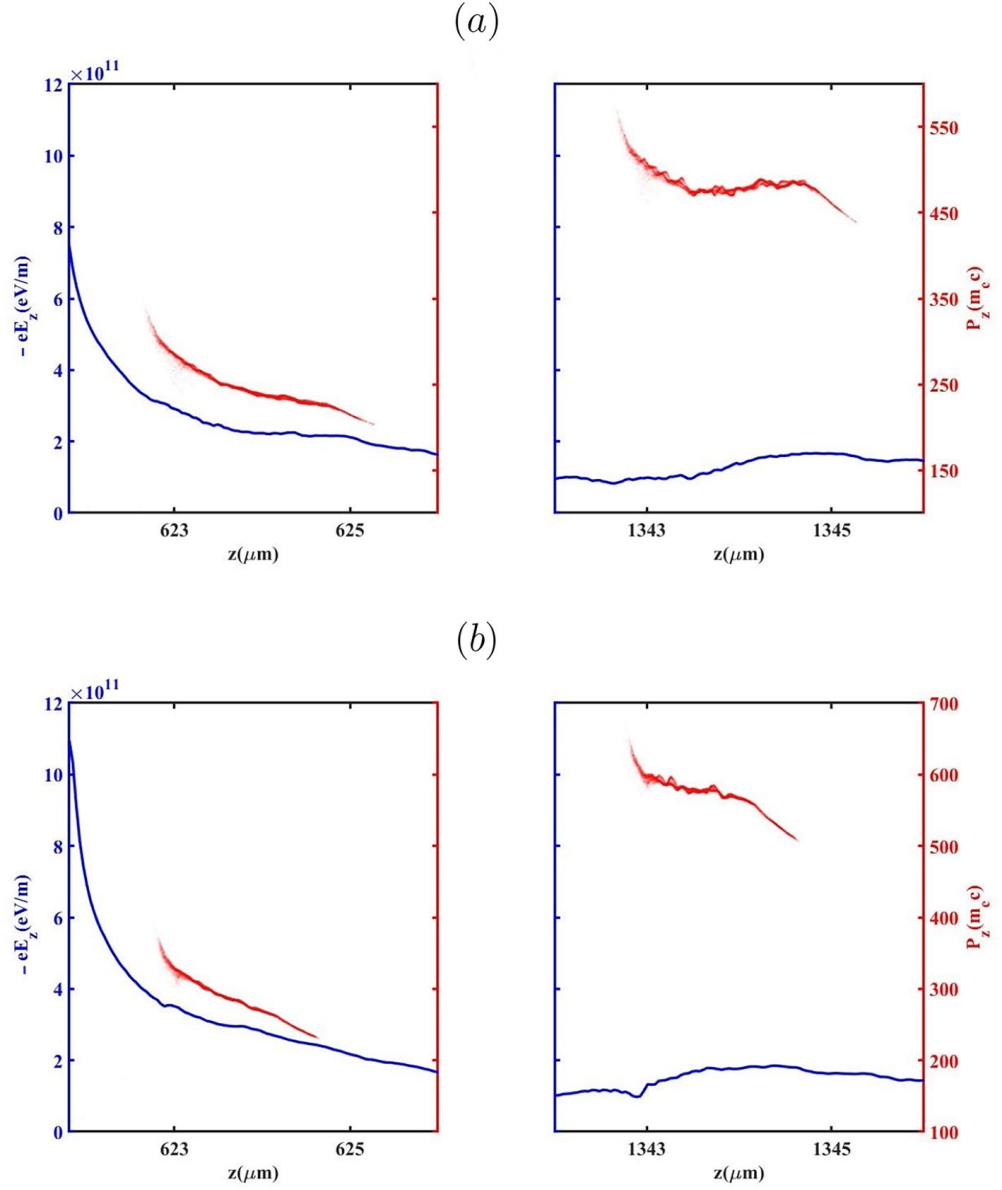
Longitudinal phase-space evolution of the injected electrons and the corresponding accelerating force ($E_z$) as a function of the bunch propagation distance for (**a**) the BG laser pulse at $L_{high} = 0 \upmu m$ and (**b**) the G laser pulse at $L_{high} = 100 \upmu m$.

**Fig. 8:**
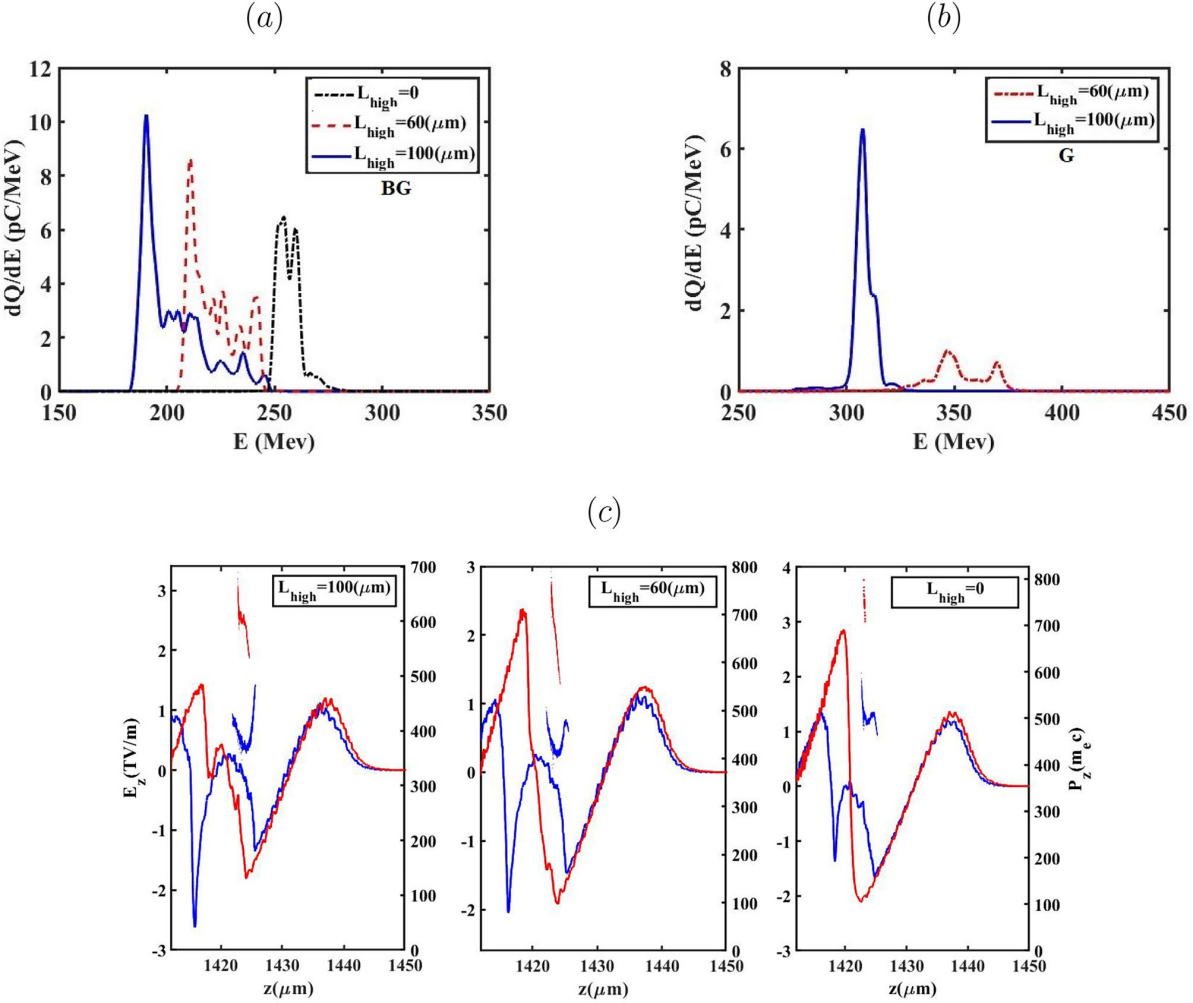
The energy spectra of injected electron bunches generated by the BG (**a**) and G (**b**) laser pulses, (**c**) corresponding phase-space distributions, overlaid with the longitudinal on-axis electric field ($E_{z}$) are presented for the BG (blue) and G (red) laser pulses.

So far, the evolution of the bubble has been examined to more clearly distinguish the effects of the BG and G laser pulses on the process of down-ramp injection. From this point forward, we focus on key properties of the electron bunch, including the evolution of RMS energy spread, emittance and energy, for the two aforementioned laser pulses. The evolution of the energy spread and mean energy for all cases of ${L_{high}}$ during the BG and G propagation as a function of the electron bunch propagation distance are presented in Fig. 6. As indicated for both laser pulses, the mean energy exhibits a monotonic increase with a reduction in ${L_{high}}$ from 100 $\upmu m$ to 0. This is attributed to a weaker beam loading effect for ${L_{high}}$=0, which yields the lowest injected charge among all considered values of ${L_{high}}$. A higher ${a_0}$ leads to the electrons having different energies being injected into the bubble. For the case of ${L_{high}}=100 \upmu m$, during the BG laser pulse propagation, the electrons injected at earlier stages are positioned at the head of the bunch and consequently acquire higher energies due to experiencing the accelerating field for the longest time. After the bubble configuration reaches its final size and electron bunches begin accelerating within the bubble, the weaker accelerating field influences the electrons positioned at the tail of the bunch. This is in contrast to the case of ${L_{high}}=0$, where weaker beam loading results in a stronger accelerating field across the bunch. As shown in Fig. 6(a), This trend manifests in the lowest energy spread observed for the ${L_{high}}=100 \upmu m$ relative to other cases, up to the electron bunch position at $z \approx 900\upmu m$. It is evident that the variation of the energy spread in the case of ${L_{high}}=60 \upmu m$ is more than other cases as the G laser pulse propagates. As previously discussed, during the G laser pulse propagation, the electrons with sufficiently high velocities are able to be injected into the bubble, particularly in the cases of ${L_{high}}=60 \upmu m$ and more prominently ${L_{high}}=0$, resulting in a reduced amount of injected charge. Therefore, a large portion of the electrons located at the tail of the bunch experiences a stronger and more rapidly increasing accelerating field relative to the case of ${L_{high}}=100 \upmu m$ due to a less-pronounced beam loading effect observed for the ${L_{high}}=60 \upmu m$. In the case of ${L_{high}}=0$, the energy spread remains nearly conserved, owing to the negligible beam loading effect caused by the very low injected charge. It should be noted that in Fig. 7 the presented cases correspond to the optimal operating conditions for each laser pulse. As demonstrated in the following sections, the optimal injection and beam quality for the G and BG pulses occur for different values of $L_{high}$ due to their distinct wake evolution and injection dynamics. To gain deeper insight into the role of the accelerating force (${\mathrm{F}}_{\mathrm{acc}}{\mathrm{=- e}}{\mathrm{E}}_{\mathrm{z}}$) in shaping the energy spread of the injected electron bunch, Fig. 7a,b illustrate the evolution of the accelerating field and the longitudinal phase space during the propagation of the BG and G laser pulses through the plasma in the cases of ${L_{high}}=0$ and ${L_{high}}=100 \upmu m$, respectively. Following the injection into the bubble, the tail of the electron bunch resides in a zone of the wakefield where accelerating force exceeds that at the head, leading to higher momentum for the electrons at the tail compared to those at the head of the bunch (called negative chirp). Over the course of a few millimeters of the propagation, the bunch’s self-loading intensifies, driven by the laser defocusing effect. As a result, the accelerating force weakens, and the self-loaded wakefield generated by the bunch becomes more prominent, leading to a flattened field profile across the bunch. This allows a substantial segment of the electrons acquires nearly the same energy, owing to their exposure to almost identical accelerating forces throughout the acceleration process. The phase-space and energy spectra of the injected electron bunches generated by the BG and G laser pulses, obtained for various ${L_{high}}$ values are presented in Fig. 8 for both BG and G laser pulses. The results indicate that, at ${L_{high}}=0, 60$ and $100 \upmu m$ electron bunches with charges of 146.57, 122.92, and 79.4 pC are injected for the BG laser pulse, while the corresponding charges for the G laser pulse are 52.4, 20.80, and  0.2 pC. The comparison reveals that electron bunches generated by the G laser pulse reach higher energies than those produced with the BG laser pulse. This is because the higher charge in the BG case loads the wake more, as indicated in Fig. 8a–c, thereby reducing the net accelerating field experienced by most of the electrons.

**Fig. 9:**
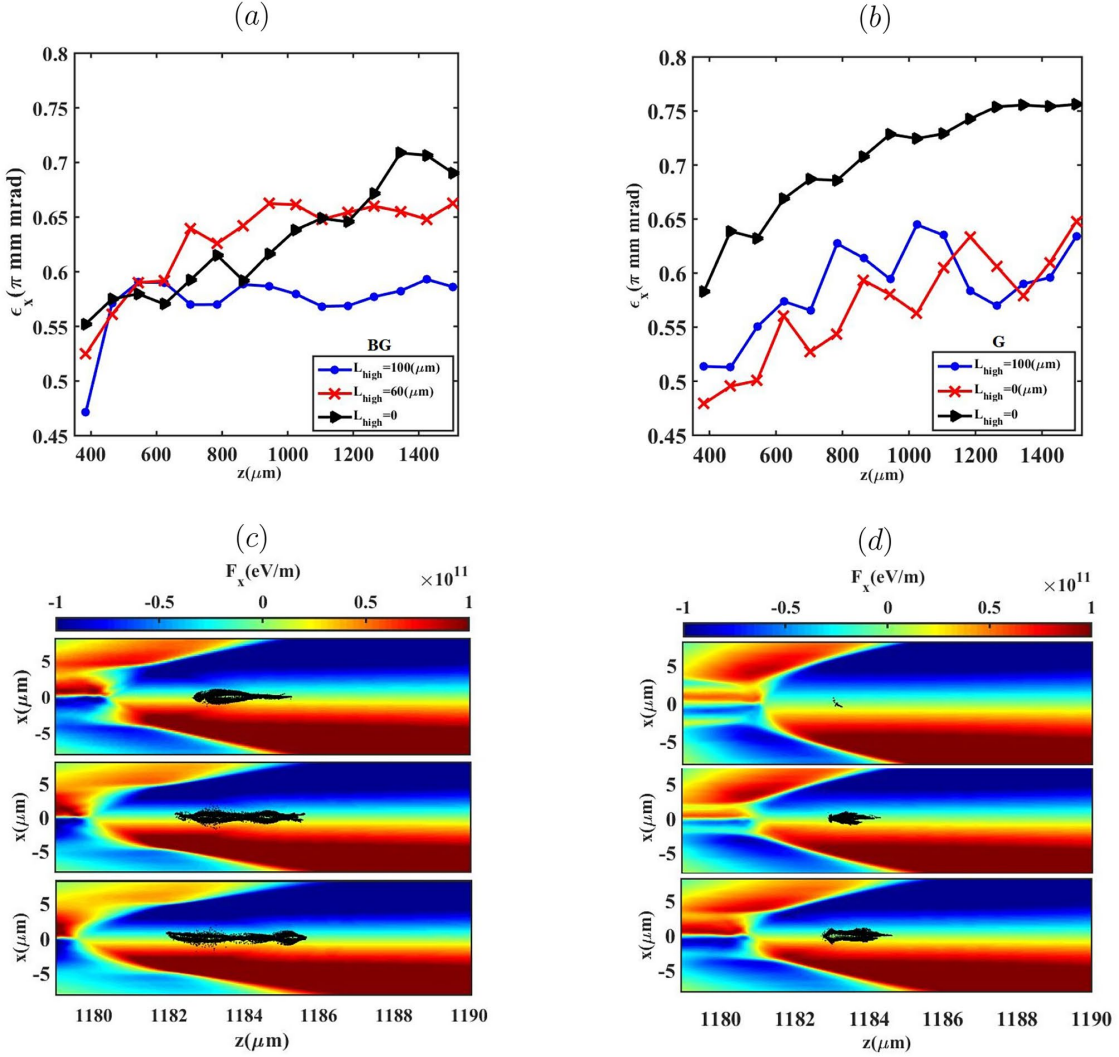
Bunch emittance evolution and transverse Lorentz force distribution ($F_x$) in the x-z plane for three lengths of ${L_{high}}=0$, $60 \upmu m$ and $100 \upmu m$ (top to bottom), for the BG (**a**,**c**) and G (**b**,**d**) laser pulses.

Figure 9 presents the evolution of the normalized emittance and the transverse Lorentz focusing force $F_x$ for different values of the high-density plateau length $L_{\text {high}}$ during the propagation of the BG and G laser pulses. The results confirm that tailoring the longitudinal plasma-density profile provides an effective means of controlling beam quality and transverse emittance. Variations in $L_{\text {high}}$ strongly influence the electron injection process, the initial transverse dynamics, and consequently the emittance evolution. For $L_{\text {high}} = 100~\mu \text {m}$, both BG and G pulses generate electron bunches that remain tightly confined throughout acceleration, exhibiting lower normalized emittance and a flatter emittance evolution compared to cases with shorter plateaus. This behavior results from earlier electron trapping induced by the extended high-density region, which favors injection from smaller transverse radii and reduces the initial betatron amplitudes, leading to a more compact transverse phase-space distribution. In contrast, for $L_{\text {high}} = 0$, electron injection occurs progressively later along the density down-ramp and from larger transverse radii. Under these conditions, electrons acquire higher transverse momentum during trapping and subsequently undergo betatron oscillations with larger amplitudes, which broadens the transverse phase-space distribution and enhances emittance growth during propagation.

**Fig. 10:**
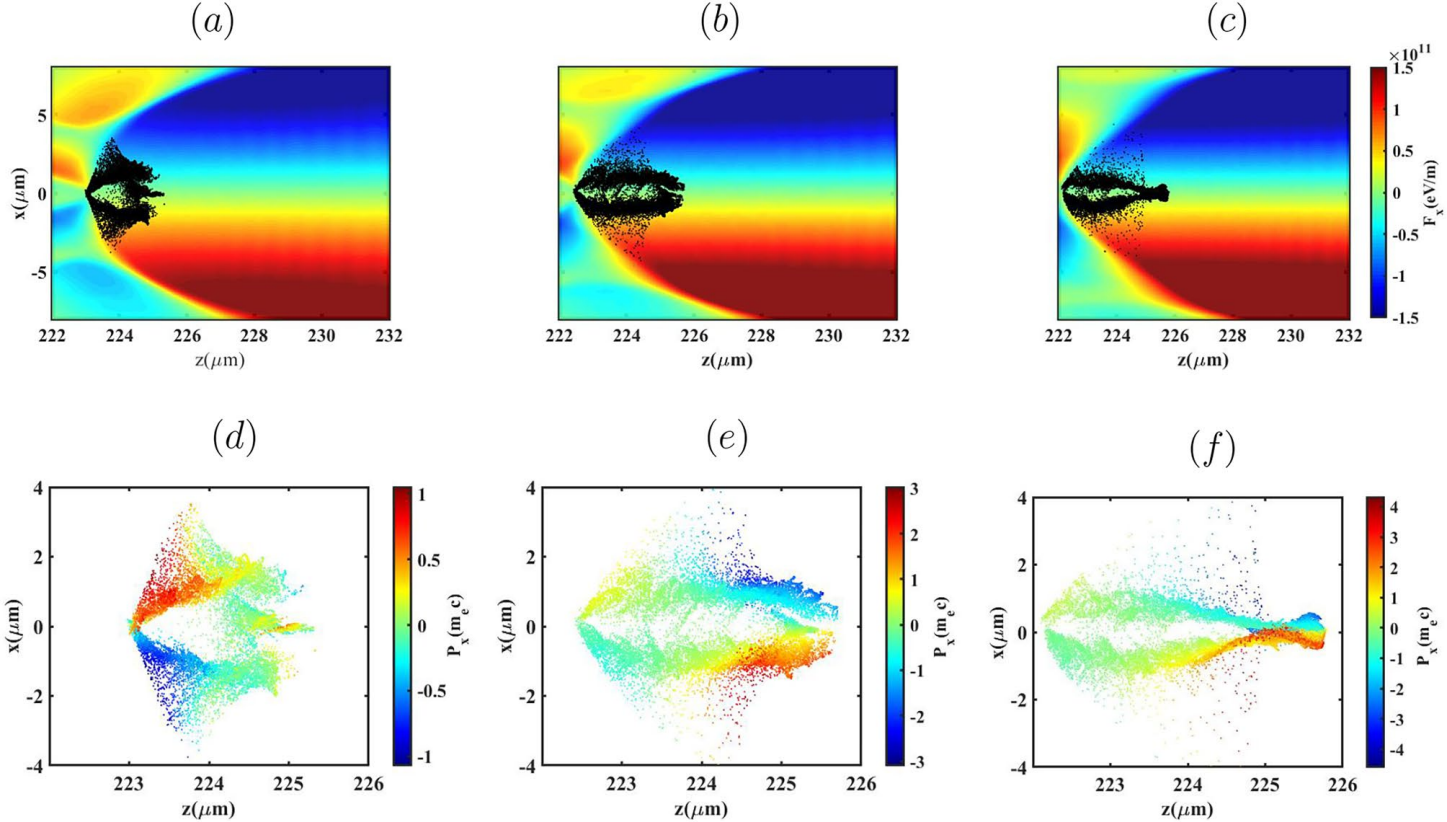
(**a**)–(**c**) Phase-space distributions of the electron bunches and (**d**)–(**f**) the corresponding transverse Lorentz force ($F_x$) for the BG laser pulse, shown for ${L_{high}} = 0~\upmu \mathrm{m}$, $60~\upmu \mathrm{m}$, and $100~\upmu \mathrm{m}$, respectively.

**Fig. 11:**
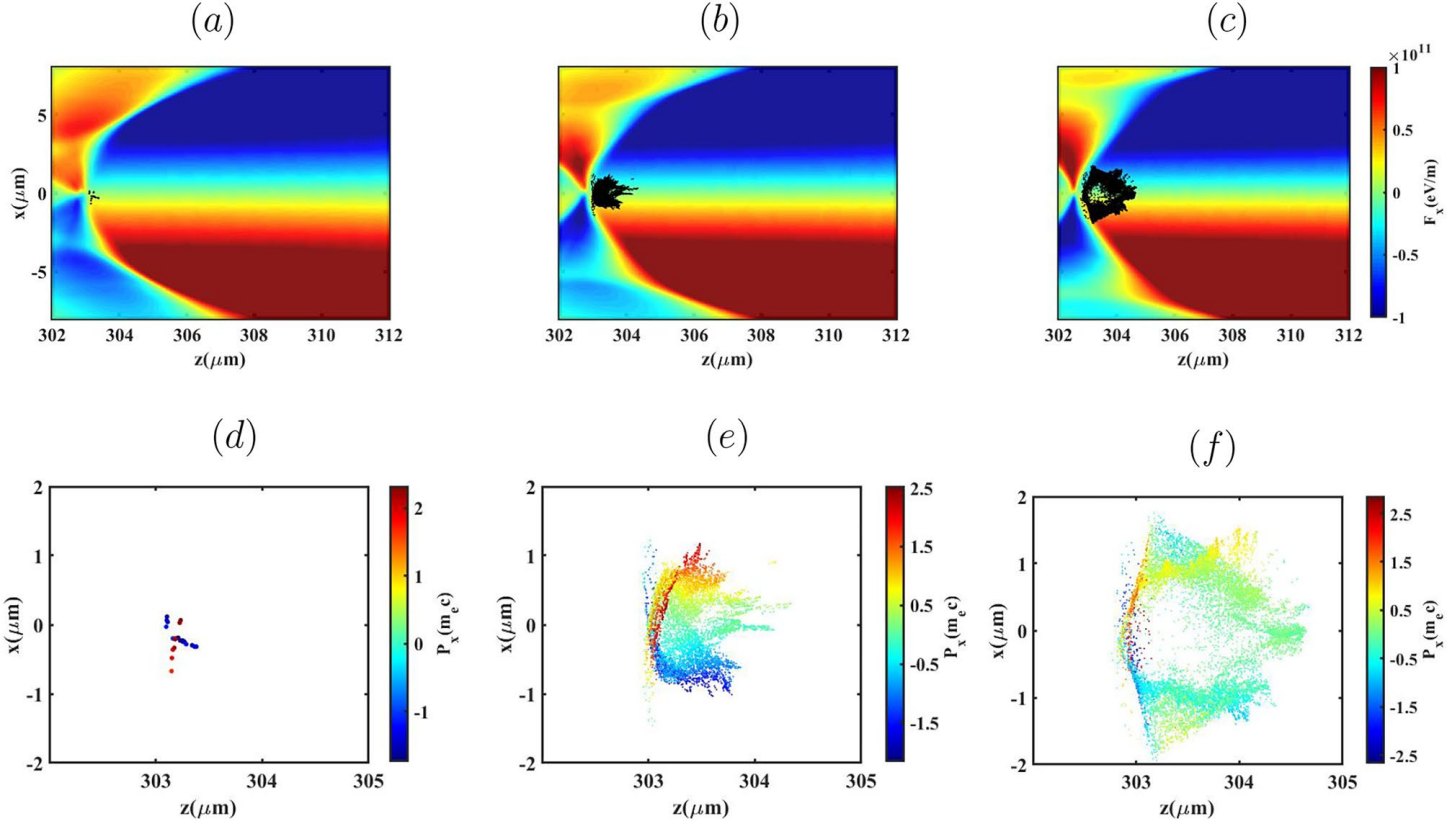
(**a**)–(**c**) Phase-space distributions of the electron bunches and (**d**)–(**f**) the corresponding transverse Lorentz force ($F_x$) for the G laser pulse, shown for ${L_{high}} = 0~\upmu \mathrm{m}$, $60~\upmu \mathrm{m}$, and $100~\upmu \mathrm{m}$, respectively.

As shown in the bottom panels of Fig. 9, the transverse Lorentz focusing force, $F_x = -e(E_x - cB_y)$, acts as an approximately linear restoring force inside the ion cavity throughout the blowout regime and does not exhibit significant variation among the different cases, in agreement with nonlinear blowout theory ^53,54^. The observed differences in emittance evolution therefore arise primarily from variations in injection timing, injection radius, and the resulting betatron dynamics, rather than from changes in the focusing strength itself. Beyond the dependence on $L_{\text {high}}$, Fig. 9 further reveals a systematic distinction between the BG and G laser drivers in terms of emittance stability, most pronounced for $L_{\text {high}} = 100~\mu \text {m}$. This difference is attributed to variations in the wakefield structure and the temporal localization of injection, which result in a higher degree of coherence in the initial betatron-phase distribution of the injected electrons. Consequently, transverse phase mixing is suppressed, and a compact phase-space distribution is preserved during propagation, sustaining a low and stable normalized emittance. For the G laser pulse, a similar qualitative dependence on $L_{\text {high}}$ is observed, although distinct features emerge in the phase-space evolution. In particular, for $L_{\text {high}} = 60~\mu \text {m}$, trailing electrons experience a stronger accelerating field owing to reduced beam loading compared with the $L_{\text {high}} = 100~\mu \text {m}$ case, allowing their trajectories to remain more closely aligned with the *z*-axis and limiting transverse divergence. In contrast, for $L_{\text {high}} = 0$, the bunch approaches transverse matching to the wake focusing channel, sustaining betatron oscillations over extended propagation and leading to a gradual increase in normalized emittance. The presence of electrons with relatively large transverse momentum near the propagation axis does not imply an increase in transverse emittance. In a linear focusing channel, betatron oscillations naturally lead to maximum transverse momentum near the axis and minimum momentum at larger transverse offsets. Consequently, the *x*–*z* distributions shown in Figs. 10 and 11 are used to visualize the spatial localization of injected electrons and their corresponding injection radii, whereas the transverse emittance is rigorously evaluated from the full transverse phase-space distribution $(x,p_x/p_z)$ and summarized in Fig. 9.

To further illustrate these transverse dynamics, Figs. 10 and 11a–f present the transverse Lorentz force ($F_x$) together with the electron distributions in the *x*–*z* plane, color-coded by their transverse momentum $p_x$, at the stage where injection has terminated and the bunches are fully trapped in the wake following the density down-ramp.

For both laser pulses, increasing $L_{\text {high}}$ modifies the injection dynamics. In the absence of an initial high-density plateau ($L_{\text {high}}=0$), the wake continues to expand during the down-ramp, enabling progressively later injection from larger transverse radii. Electrons injected from these larger radii are pulled toward the axis during trapping, which bends their trajectories and increases their transverse momentum $p_x$, as expected for betatron motion in a linear focusing channel. Because these electrons are injected later and with larger initial betatron amplitudes, their transverse phase-space distribution becomes broader, leading to the higher emittance observed in Fig. 9 for the $L_{\text {high}}=0$ case. Conversely, for $L_{\text {high}}=100~\mu \text {m}$, injection occurs earlier and over a shorter time interval, before the wake undergoes significant radial expansion in the down-ramp region. Consequently, electrons are injected closer to the axis with smaller initial transverse momenta and larger longitudinal momenta, resulting in smaller betatron amplitudes. This yields a more compact transverse phase-space distribution and the lower, more stable emittance shown in Fig. 9.

It is important to note that the presence of electrons with relatively high $p_x$ near the axis in the $L_{\text {high}}=100~\mu \text {m}$ case does not contradict the lower emittance. In the blowout regime the transverse Lorentz force is linear and restoring, so electrons undergoing betatron motion naturally reach their maximum $p_x$ near the axis and minimum $p_x$ at larger radii. For $L_{\text {high}}=100~\mu \text {m}$, the early, short-duration injection rapidly confines the electrons to the near-axis region where the focusing fields are predominantly linear. The resulting increase in $p_x$ therefore corresponds to a correlated rotation of the transverse phase space rather than phase-space dilution, which preserves a small emittance. A similar qualitative behavior is observed for the G laser pulse, where the differences in emittance arise primarily from variations in injection radius and timing, not from a change in the transverse focusing force, which remains linear and approximately uniform along the bunch in the blowout regime.

**Table 1:** Tabulation of electron bunch parameters for different $L_{high}$: the injected bunch charge (Q), Mean energy $(\mathop E\nolimits _{mean })$, energy spread $(\Delta E/E \%)$ and transverse normalized emittance $(\mathop \varepsilon \nolimits _x - \mathop \varepsilon \nolimits _y)$.

Laser Pulse	$L_{high}$ ($\mu$m)	Q (pC)	$\mathop E\nolimits _{mean }$ (MeV)	$\Delta E/E$ (%)	$\mathop \varepsilon \nolimits _x - \mathop \varepsilon \nolimits _y$ ($\pi$ mm mrad)
BG	0	79.4	263	2.3	0.69-0.18
	60	122.92	225.57	6	0.66-0.21
	100	146.57	205.87	8.3	0.58-0.24
G	0	0.2	397.70	2.4	0.75-0.12
	60	20.8	363.98	3.4	0.64-0.2
	100	52.40	317.24	1.3	0.63-0.16

As quantitatively summarized in Table 1, the influence of the initial plasma density gradient (${L_{high}}$) on the injected electron bunch parameters is pronounced. While increasing ${L_{high}}$ from 0 to 100 $\upmu m$ successfully enhances the trapped charge–reaching a maximum of 146.57 pC for the BG pulse–this enhancement comes at a significant degradation of beam quality, evidenced by the energy spread ($\Delta E/E$) rising from 2.3% to 8.3%. Critically, the data unequivocally supports the primary finding of this work: the optimal regime for phase-space purity is strictly defined by ${L_{high}}=0$. Under this condition, the G pulse maintains an exceptionally low energy spread of 2.4% at a mean energy of 397.70 MeV, confirming that minimizing beam loading effects via optimized temporal injection timing (${L_{high}}=0$) overrides the benefit of higher charge accumulation at non-ideal timings. Therefore, the most critical criterion for producing high-quality electron beams is precise temporal control over the injection threshold, as facilitated by the ${L_{high}}=0$ profile geometry.

**Fig. 12:**
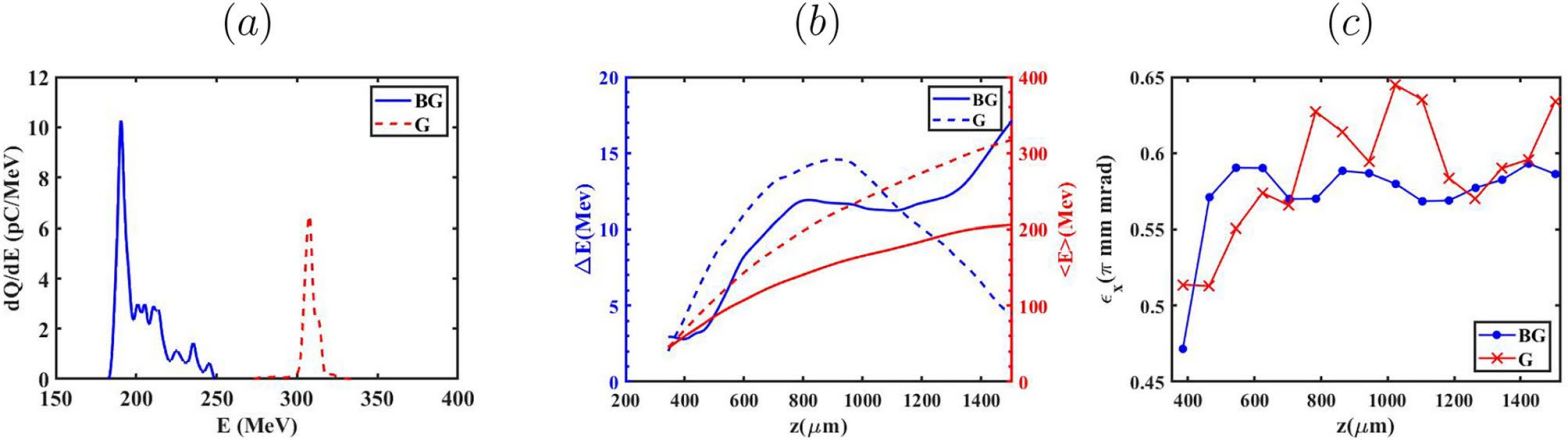
Comparison of beam quality parameters as a function of the propagation distance *z* for BG and G laser pulses, performed at equal total laser energy and for $L_{high}=100~\mu$m. (**a**) Energy spectra of the accelerated electron bunches. (**b**) Evolution of the mean electron energy $\langle E \rangle$ (right axis) and absolute energy spread $\Delta E$ (left axis). (**c**) Normalized transverse emittance $\varepsilon _x$ evolution along the propagation direction.

In summary, this study has demonstrated that longitudinal plasma density tailoring provides a robust and flexible means of regulating electron injection and beam quality in laser wakefield acceleration driven by different transverse laser profiles. By adjusting the ramp parameter $L_{high}$, the onset, duration, and spatial extent of injection can be systematically tuned, thereby enabling controlled modification of charge loading, energy spread, and transverse phase–space evolution. Under the constraint of fixed total laser energy, the two transverse laser profiles examined here promote distinct beam characteristics. BG pulses, owing to their transverse intensity distribution and the resulting wake structure, tend to support enhanced charge injection, whereas G pulses favor the generation of electron bunches with higher mean energies and comparatively lower relative energy spread. When combined with plasma-density tailoring, these pulse-dependent trends are consistently observed across the investigated configurations and are associated with differences in injection dynamics, rather than with arbitrary or case-specific parameter choices. Taken together, these findings highlight the importance of jointly tailoring the plasma-density profile and the laser transverse structure to achieve controlled and application-relevant optimization of electron-beam properties. Figure 12 provides a compact comparison of these beam characteristics under the fixed-energy condition, summarizing the trends discussed above.

## Conclusions

In this work, a systematic PIC-based investigation was conducted to examine how laser pulse shaping and longitudinal plasma density tailoring jointly influence electron injection dynamics and the resulting beam properties in laser wakefield acceleration. By comparing Gaussian (G) and zeroth-order Bessel–Gaussian (BG) drivers under identical total laser-energy conditions and across multi-section plasma profiles, the respective roles of laser structure and density geometry in determining charge, energy, and transverse beam quality were clarified in a unified framework. The results demonstrate that BG pulses consistently support extended or repeated injection episodes, yielding higher trapped charge across all tested $L_{\mathrm{high}}$ values. This behavior is primarily attributed to their enhanced self-focusing and modified wake excitation. Conversely, the G pulse promotes more localized injection with reduced beam loading, producing higher peak energies and improved spectral purity, particularly for $L_{\mathrm{high}} = 0$, where a relative energy spread of 2.4% was obtained. The parameter $L_{\mathrm{high}}$ was shown to serve as a robust and practical control knob for regulating the onset, duration, and amplitude of injection events in both laser configurations, thereby enabling predictable charge–energy trade-offs. Although no fundamental difference in transverse emittance trends was observed between the BG and G pulses under identical plasma conditions, the injection timing and spatial localization governed by $L_{\mathrm{high}}$ significantly influenced the coherence of transverse motion. In particular, stable and low emittance was maintained when the initial betatron-phase distribution remained compact during propagation, as observed for BG injection at $L_{\mathrm{high}} = 100~\upmu \mathrm{m}$.

Overall, this study highlights that combining moderate laser-pulse shaping with tailored plasma-density profiles provides a flexible and experimentally accessible approach for controlling beam characteristics in LWFA. Rather than positioning BG and G pulses as competing options, the results indicate that they offer complementary pathways for tuning beam loading, charge, and final energy. This synthesis of laser profile engineering and density tailoring provides actionable design insight for optimizing accelerator performance across different operating objectives.

## Data Availability

The data that support the findings of this study are available from the corresponding author upon reasonable request.
